# Retrospective Analysis of a Quince, Olive Leaf, and Amaranth Nutraceutical in Patients with Metabolic Syndrome

**DOI:** 10.3390/medicina61091638

**Published:** 2025-09-10

**Authors:** Luigi Sardelli, Anna Esposito, Antonio De Mitri, Nunzia Fele, Fabio Turco, Vincenzo Desiderio, Luigi Pulcrano

**Affiliations:** 1 Centro Antidiabete Ds 51, ASL Napoli 3 Sud, 80038 Pomigliano d’Arco, Italy; luigisardelli@libero.it (L.S.); aryespy@gmail.com (A.E.); antoniodemitri91@gmail.com (A.D.M.); luigi_pulcrano@libero.it (L.P.); 2 U.O.C. Cardiologia, AORN Ospedali dei Colli, Ospedale Monaldi, 80131 Naples, Italy; nunzia-80@libero.it; 3 Independent Researcher, Via della Veterinaria, 80137 Naples, Italy; fabio.turco81@yahoo.it; 4 Department of Experimental Medicine, University of Campania “Luigi Vanvitelli”, 80138 Naples, Italy

**Keywords:** metabolic syndrome, *Amaranthus*, *Cydonia oblonga*, olive leaf, diabetes mellitus, nutraceuticals, dyslipidemia, insulin resistance, cardiovascular health, real-world evidence

## Abstract

*Background and Objectives*: Metabolic syndrome (MetS) is characterized by a cluster of factors, including dyslipidemia, insulin resistance, central obesity, elevated blood pressure, and impaired fasting glucose, which together elevate the risk of type 2 diabetes and cardiovascular disease. Nutraceuticals containing botanical extracts with antioxidant and metabolic activity have emerged as promising adjunctive strategies in the management of MetS. This study evaluated the clinical effectiveness and biological rationale of a standardized food supplement (QUINOLAM), containing extracts of *Cydonia oblonga* (quince), *Olea europaea* (olive leaf), and *Amaranthus* spp., in adults with metabolic syndrome. *Materials and Methods*: This was a retrospective, single-center observational study including adults with documented MetS who received one tablet daily of the QUINOLAM-based supplement for at least 12 weeks. The primary endpoint was the change in total cholesterol. Secondary endpoints included LDL-C, HDL-C, triglycerides, fasting glucose, HbA1c, HOMA-IR, CRP, and BMI. In parallel, preclinical studies were conducted using HepG2 cells to investigate QUINOLAM’s effects on LDL receptor expression, glucose uptake, antioxidant activity, and cell viability. *Results*: Thirty patients met the inclusion criteria. A significant reduction in total cholesterol was observed at both 6 and 12 weeks (*p* < 0.005), accompanied by a significant decline in LDL-C by week 12 (*p* < 0.05). Among patients with baseline fasting glucose ≥100 mg/dL (*n* = 19), a significant improvement in glycemia was recorded (*p* < 0.005). Trends toward improvement were noted in other metabolic indices. In vitro, QUINOLAM enhanced LDL receptor expression (*p* < 0.05) and glucose uptake (*p* < 0.01), demonstrated antioxidant activity in the TEAC assay, and showed no cytotoxicity at relevant doses. *Conclusions*: In a real-world setting, daily supplementation with QUINOLAM was associated with significant improvements in lipid and glycemic control among patients with MetS. Preclinical findings further support its mechanistic plausibility via modulation of LDL handling, glucose metabolism, and oxidative stress. These results warrant confirmation in larger, prospective clinical trials.

## 1. Introduction

Metabolic syndrome (MetS) is a multifactorial disorder defined by a cluster of interrelated risk factors, including obesity, dyslipidemia, insulin resistance, and elevated blood pressure [1]. Together, these abnormalities markedly increase the likelihood of developing type 2 diabetes mellitus, atherosclerotic cardiovascular disease, and premature mortality [2]. The prevalence of MetS has risen globally in parallel with the increased consumption of hypercaloric, nutrient-poor diets and sedentary lifestyles, particularly in urban populations of both developed and developing countries [3]. Considering only the European adult population, MetS prevalence is estimated at around 20–25%, reflecting a substantial public health burden [4]. Italy exemplifies this impact, with recent reports indicating approximately 19.6% of Italian men and 33.3% of women meet MetS criteria [5].

Among the pathophysiological mechanisms underpinning MetS, insulin resistance plays a central role [2]. It contributes to altered glucose and lipid metabolism, chronic low-grade inflammation, and neurohormonal dysregulation. Although lifestyle modification remains the foundation of MetS management, long-term adherence is often suboptimal [4]. Accordingly, there is increasing interest in evidence-based nutraceutical strategies that may complement traditional approaches. Among such interventions, plant-derived compounds with antioxidant, anti-inflammatory, and metabolic-modulating properties are under active investigation. Extracts from *Cydonia oblonga* (quince), *Olea europaea* (olive), and *Amaranthus* spp., leaves have attracted particular attention due to their bioactive profiles and traditional medicinal use in the context of metabolic and cardiovascular disorders.

Quince (*Cydonia oblonga*) and its derivatives (leaf, seed, fruit) are high in polyphenols, organic acids, and flavonoids with antioxidant, anti-inflammatory, hypoglycemic, and lipid-lowering properties [5]. A systematic review of animal and in vitro studies reports quince effects on lowering blood pressure, improving glucose metabolism, and normalizing lipid profiles, with magnitudes comparable to captopril (for hypertension) and simvastatin (for dyslipidemia) [6]. In diabetic rat models, aqueous extract of quince fruit significantly lowered serum triglycerides and total cholesterol, while improving liver and renal parameters [7]. Quince also demonstrated hepatoprotective and cardioprotective effects in models of toxin-induced injury [8,9,10,11]. Phytochemical profiling confirms high concentrations of carotenoids, polyphenols, and vitamin C, supporting its metabolic efficacy [12].

Olive leaf extract, obtained from *Olea europaea* leaves, is another promising nutraceutical candidate for cardiometabolic health [13]. Extracts from olive leaves are particularly rich in phenolic secoiridoids such as oleuropein, hydroxytyrosol, and verbascoside, compounds with potent antioxidant, anti-inflammatory, and vasoprotective activity [14,15]. Preclinical studies have demonstrated that olive leaf extract supplementation improves lipid profiles, reduces insulin resistance, and restores hepatic antioxidant gene expression in rodent models of aging and metabolic dysfunction [16,17]. Notably, aged rodents given olive leaf extract showed lowered LDL cholesterol and inflammatory cytokines (IL-6), alongside preserved endothelial function [18]. Clinically, randomized controlled trials (RCTs) have reported that OLE supplementation improves insulin sensitivity and glycemic control in overweight and prediabetic individuals, reduces blood pressure in hypertensive patients, and favorably modulates lipid profiles [19,20,21].

Amaranth (*Amaranthus* spp.) is a pseudocereal rich in polyunsaturated fatty acids, squalene, tocopherols, and phytosterols [22]. Preclinical studies have shown that amaranth supplementation improves glycemic control, reduces serum and hepatic lipid levels, and enhances antioxidant defenses. In animal models of streptozotocin-induced diabetes, supplementation with amaranth grain or seed oil significantly improved glucose tolerance, reduced fasting blood glucose, and lowered serum and hepatic triglycerides and total cholesterol [23]. Similar hypolipidemic and antioxidant effects were observed across various Amaranthus species, improving hepatic steatosis, boosting endogenous antioxidant systems, and attenuating obesity-induced metabolic dysfunctions [24]. In human studies, amaranth supplementation has been shown to improve key metabolic parameters, including glycemic control and lipid profiles [25]. Trials in cardiovascular disease patients also showed that amaranth oil lowered blood pressure and improved lipid profiles [26].

Given the distinct but complementary actions of quince, olive leaf, and amaranth on key metabolic pathways, including synergistic improvements in lipid handling, glycemic control, antioxidant defense, and inflammation, a combination of these extracts may provide multi-targeted support in MetS. In fact, a standardized formulation containing quince–olive–amaranth extracts (QUINOLAM) has recently been developed to capitalize on these combined mechanisms, with the aim of supporting lipid and glucose homeostasis in individuals at elevated metabolic risk. Importantly, polyphenol-rich botanical supplements such as those in QUINOLAM generally exhibit a favorable safety profile at customary dietary or supplemental doses. For instance, a 24-week randomized trial of olive leaf extract reported that the supplement was well-tolerated with no severe adverse events compared to placebo [27]. Likewise, amaranth has been consumed as a staple food grain for millennia with no known safety concerns, and quince fruit and leaf preparations have a long history of traditional medicinal use without major adverse effects reported [12,22]. This encouraging safety and tolerability profile supports the feasibility of using QUINOLAM as an adjunct in MetS care.

Despite promising data on each of these botanicals, there remains a clinical gap regarding their combined efficacy in MetS, especially in real-world settings. To address this, the present study was designed to evaluate the metabolic impact of the QUINOLAM supplement in a real-world Italian MetS population through a retrospective observational approach. We hypothesized that daily supplementation with QUINOLAM over 12 weeks would yield improvements in lipid parameters and glycemic indices, alongside reductions in inflammatory markers and insulin resistance, reflecting the complementary mechanisms of its components. In parallel, mechanistic in vitro assays were conducted to strengthen the translational understanding of QUINOLAM’s actions on hepatic LDL receptor expression, glucose uptake, and antioxidant capacity. This combined retrospective and preclinical evaluation is novel in examining a quince–olive leaf–amaranth supplement within a routine care setting, and aims to provide preliminary evidence and mechanistic insight into the efficacy of this multi-component nutraceutical for MetS management in the Italian population.

## 2. Materials and Methods

### 2.1. Study Design

This was a retrospective, monocentric, observational, non-controlled study conducted to evaluate the effects of a standardized combination of *Cydonia oblonga*, *Olea europaea* leaves, and *Amaranthus* (QUINOLAM) extracts on metabolic parameters in individuals at elevated cardiometabolic risk, complemented by a set of preclinical in vitro assays. The retrospective study was performed in accordance with the STROBE guidelines for observational studies [28]. The clinical study was carried out at the Ambulatory Diabetology Unit, District 51, ASL Napoli 3 SUD, Italy. The study was approved by the Institutional Ethics Committee of Campania 2 (protocol code 0138743/i of 6 June 2025). Data were collected from medical records of patients who had received the QUINOLAM food supplement for at least 12 weeks as part of routine clinical practice. No compensation was provided to investigators, and no additional cost was incurred by the healthcare system. All patients signed an informed consent form prior to inclusion. All procedures adhered to ethical standards set by the responsible committee on human experimentation (both institutional and national), as well as the Helsinki Declaration of 1975, as revised in 2008. Preclinical in vitro studies were conducted in accordance with Good Laboratory Practices (GLP) by BiCT S.r.l. Laboratoris (Lodi, Italy).

### 2.2. Preclinical Study

#### 2.2.1. Chemical Characterization of QUINOLAM

The chemical composition of QUINOLAM was evaluated through quantitative and qualitative assays performed under GLP-certified laboratory conditions. The total polyphenol content and specific phenolic constituents of the formulation were quantified using high-performance liquid chromatography with photodiode array detection (HPLC-PDA). Approximately 1 g of QUINOLAM powder was extracted in 50% ethanol under agitation. The supernatant was filtered and injected into the HPLC system equipped with a reversed-phase C18 column and PDA detector. Mobile phases consisted of water with 0.1% formic acid (solvent A) and acetonitrile with 0.1% formic acid (solvent B), using a linear gradient. Detection wavelengths were set at 280 nm and 320 nm for oleuropein and hydroxytyrosol, respectively. Quantification was performed against external calibration curves of authenticated standards. The total polyphenol content was calculated as the sum of identified compounds, expressed as mg/kg of dry weight. The fiber profile of the formulation was determined by the Van Soest detergent method, which sequentially isolates fiber fractions through acid and neutral detergent extractions. Acid detergent fiber (ADF), neutral detergent fiber (NDF), cellulose, hemicellulose, and lignin were quantified gravimetrically. Analyses were conducted on dried samples of QUINOLAM using standard laboratory procedures adapted for botanical matrices.

#### 2.2.2. Cell Lines and Culture Conditions

All in vitro experiments were conducted using the HepG2 human hepatic cell line (ATCC, Manassas, VA, USA). Cells were cultured in MEM (Gibco, Thermo Fisher Sci-entific, Waltham, MA, USA) supplemented with 10% fetal bovine serum (FBS, qualified, heat-inactivated; Gibco), 1% L-glutamine (Gibco), and 1% penicillin/streptomycin (Gibco), under standard conditions (37 °C, 5% CO_2_) (Thermo Scientific Heracell™ 150i). All experiments were performed in triplicate across at least three independent biological replicates to ensure reproducibility.

#### 2.2.3. Cell Viability Assay

Cytotoxicity of QUINOLAM was assessed using the 3-(4,5-dimethylthiazol-2-yl)-2,5-diphenyltetrazolium bromide (MTT) assay. The reagent used was Thiazolyl Blue Tetrazolium Bromide (Sigma-Aldrich, Merck KGaA, Darmstadt, Germany); cells were seeded in 96-well plates and treated with 10 serial dilutions of QUINOLAM (1.6 mg/mL to 0.003 mg/mL) for 24 h. Cell viability was expressed as a percentage relative to untreated controls. Sodium dodecyl sulfate (SDS, 1 mg/mL) was used as a positive control for cytotoxicity.

#### 2.2.4. Glucose Uptake Assay

To investigate the effects of QUINOLAM on glucose metabolism, we employed the fluorescent glucose analog 2-NBDG (2-(N-(7-nitrobenz-2-oxa-1,3-diazol-4-yl)amino)-2-deoxyglucose; Thermo Fisher Scientific, Waltham, MA, USA). HepG2 cells were seeded in 96-well plates at a density of 1 × 10^4^ cells per well and allowed to adhere for 24 h under standard culture conditions. Cells were then treated with QUINOLAM at concentrations of 0.5, 1.0, and 2.5 mg/mL for 24 h in complete MEM medium. Following treatment, cells were washed twice with phosphate-buffered saline (PBS, Thermo Fisher Scientific, Waltham, MA, USA) and incubated with 100 µM 2-NBDG (Thermo Fisher Scientific, Waltham, MA, USA) in glucose-free MEM (Gibco, Thermo Fisher Scientific, Waltham, MA, USA) for 30 min at 37 °C to allow uptake of the fluorescent probe. After incubation, cells were washed to remove extracellular 2-NBDG, and fluorescence intensity was measured using a multimode plate reader (Synergy™ H1, BioTek Instruments, Winooski, VT, USA) with excitation at 465 nm and emission at 540 nm. Results were normalized to untreated controls, and all experiments were performed in triplicate across at least three independent runs to ensure reproducibility.

#### 2.2.5. LDL-Receptor Expression

The expression of low-density lipoprotein receptors (LDL-R) was quantified using an ELISA-based assay following a 24 h treatment with QUINOLAM (0.5, 1.0, and 2.5 mg/mL).

#### 2.2.6. Antioxidant Activity

The antioxidant capacity of QUINOLAM was determined by the Trolox Equivalent Antioxidant Capacity (TEAC) assay. The supplement was solubilized in water and tested at concentrations of 0.05, 0.1, and 0.2 mg/mL. Antioxidant activity was expressed as Trolox equivalents.

### 2.3. Clinical Study

#### 2.3.1. Study Population

The study involved a population of patients with elevated metabolic risk who had taken one tablet per day of the QUINOLAM food supplement for a minimum of 12 weeks. Patients were selected from a general practice setting.

##### Inclusion Criteria

Patients aged 18 years or older with documented metabolic risk were eligible if they met at least three diagnostic features of metabolic syndrome, including increased waist circumference (>102 cm in men or >88 cm in women), elevated triglycerides (≥150 mg/dL or use of lipid-lowering therapy), reduced HDL-C (<40 mg/dL in men or <50 mg/dL in women or use of therapy), elevated blood pressure (≥130/85 mmHg or antihypertensive treatment), fasting plasma glucose ≥100 mg/dL or established type 2 diabetes, HOMA-IR >2, or total cholesterol >180 mg/dL. Eligible patients also had to provide at least two biochemical assessments (baseline and after 12 weeks of supplement use) and written informed consent for retrospective analysis.

##### Exclusion Criteria

Patients were excluded if they used statins or other lipid-lowering drugs during the study period, received more than two concomitant antihypertensive or antidiabetic medications, consumed other supplements with potential lipid-lowering properties, or had missing or incomplete bio-chemical data.

#### 2.3.2. Study Procedures

Given the retrospective nature of this study, no protocol-specific visits, additional diagnostic procedures, or interventional measures were carried out. Instead, all information was derived from medical records routinely collected during standard clinical care. Patients who fulfilled the inclusion criteria were identified through chart review and subsequently contacted to obtain written informed consent for the use of their anonymized data in the analysis. Clinical and biochemical parameters were extracted from two pre-defined timepoints, namely the visit immediately preceding the initiation of the food supplement and the follow-up visits performed after 6 and 12 weeks of use. This approach ensured that data reflected real-world clinical practice without introducing additional biases related to study-specific procedures.

#### 2.3.3. Clinical Endpoints

##### Primary Endpoint

Absolute and percentage change in total cholesterol between baseline and 12 weeks of supplementation.

##### Secondary Endpoints

Absolute and percentage changes were assessed between baseline, 6 weeks, and 12 weeks for several key clinical parameters. These included low-density lipoprotein cholesterol (LDL-C), high-density lipoprotein cholesterol (HDL-C), triglycerides, fasting blood glucose, and glycated hemoglobin (HbA1c). In addition, variations in the Homeostasis Model Assessment of Insulin Resistance (HOMA-IR) index, C-reactive protein (CRP), and body mass index (BMI) were also calculated to provide a comprehensive evaluation of metabolic and inflammatory status over time.

#### 2.3.4. Data Collection

Pseudonymized clinical and laboratory data were extracted from the institutional database using a standardized case report form (CRF). Parameters collected included age, anthropometric measures, blood pressure, lipid profile, glycemic indices, HOMA-IR index, and CRP. All data were stored on secure servers with restricted access, in compliance with General Data Protection Regulation (GDPR) regulations.

#### 2.3.5. Intervention

The intervention consisted of the routine administration of a commercially available food supplement, containing aqueous extracts of QUINOLAM, taken as one tablet per day for a minimum duration of 12 weeks. The supplement was taken without changes to background therapies, except where specified in the exclusion criteria. The dosage and administration schedule were recorded according to real-world usage.

#### 2.3.6. Food Supplement

Novametabolic^®^ (Nova Argentia, Gorgonzola, Italy) is a dietary supplement composed of standardized extracts from *Cydonia oblonga* (quince), *Olea europaea* (olive leaf), and *Amaranthus* spp., collectively referred to as QUINOLAM in this paper. The nutraceutical formulation contains the following: Cydonia oblonga fruit extract: 20–30%; Olea europaea leaf extract: 20–25%; Amaranthus caudatus seed extract: 25–30%. It has been authorized by the Italian Ministry of Health and is marketed in compliance with national food supplement regulations. This formulation was specifically selected for its complementary metabolic, antioxidant, and anti-inflammatory properties. The active components include polyphenols, flavonoids, and organic acids (from quince); secoiridoids such as oleuropein and hydroxytyrosol (from olive leaf); and unsaturated fatty acids, phytosterols, squalene, and tocopherols (from amaranth).

#### 2.3.7. Statistical Analysis

All statistical analyses were conducted using non-parametric methods, consistent with the study’s observational design and small sample size. The Wilcoxon signed-rank test was applied for paired comparisons between baseline and 12-week values. The Friedman test was used to evaluate repeated measures across three timepoints (baseline, 6 weeks, and 12 weeks), when available. Missing data were handled by complete case analysis, meaning only participants with data at all relevant timepoints were included in the respective analyses. In line with the pre-specified analysis plan, fasting glucose was also analyzed as a secondary outcome in the subgroup of patients with baseline values ≥100 mg/dL. A two-tailed *p*-value < 0.05 was considered statistically significant. All analyses were conducted using GraphPad Prism (version 10).

## 3. Results

### 3.1. Preclinical Data

#### 3.1.1. Chemical Characterization of QUINOLAM

The phytochemical profile of QUINOLAM confirmed a high content of bioactive polyphenols and dietary fiber. HPLC-PDA analysis revealed a total polyphenol concentration of 77,137 mg/kg, with oleuropein and hydroxytyrosol quantified at 25,315 mg/kg and 3117 mg/kg, respectively. These values indicate a significant contribution from Olea europaea leaf extract, consistent with the expected formulation composition. Fiber analysis using the Van Soest method showed an ADF content of 27.2%, a NDF content of 32.4%, with cellulose (17.0%), hemicellulose (5.2%), and lignin (10.2%) contributing to the overall dietary fiber matrix.

#### 3.1.2. Cell Viability Assay

**Figure 1:**
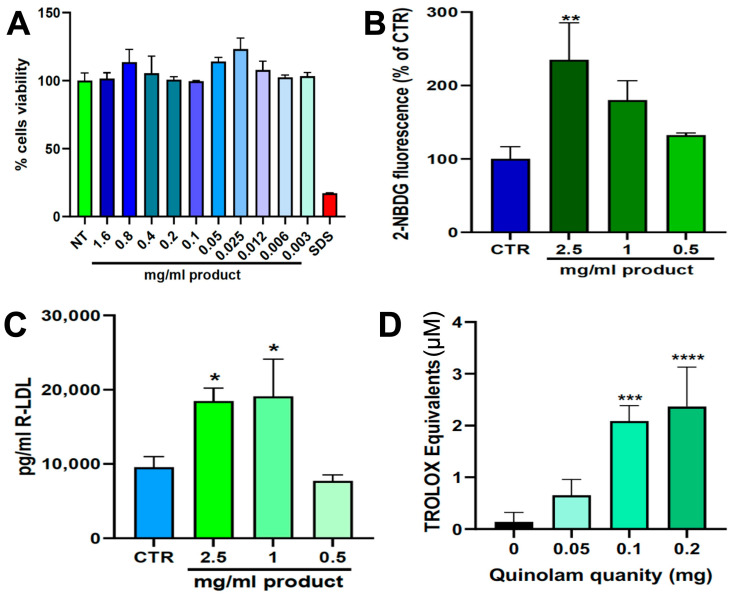
Preclinical evaluation of QUINOLAM on metabolic and cellular endpoints in HepG2 cells. (**A**) Cell viability assessed by MTT assay after 24 h exposure to increasing concentrations of QUINOLAM (0.003 to 1.6 mg/mL). No cytotoxic effects were observed up to 1.6 mg/mL. SDS (1 mg/mL) was used as a positive control. (**B**) Glucose uptake measured using the 2-NBDG fluorescent analog following 24 h treatment with QUINOLAM at 0.5, 1.0, and 2.5 mg/mL. A dose-dependent increase in glucose uptake was observed, with significant enhancement at 2.5 mg/mL (*p* < 0.01 vs. control). (**C**) LDL receptor (LDL-R) protein expression determined by ELISA after 24 h exposure to QUINOLAM. Treatments with 1.0 and 2.5 mg/mL significantly upregulated LDL-R levels compared to untreated controls (*p* < 0.05). (**D**) Antioxidant activity of QUINOLAM evaluated using the Trolox Equivalent Antioxidant Capacity (TEAC) assay. QUINOLAM displayed strong antioxidant potential in a dose-dependent manner, with significant increases in TEAC values at 0.1 and 0.2 mg/mL (* *p* < 0.05, ** *p* < 0.01, *** *p* < 0.001, **** *p* < 0.0001 vs. control). All data are expressed as mean ± standard deviation from three independent experiments.

QUINOLAM demonstrated an excellent safety profile in HepG2 cells. Across the tested concentration range (0.003–1.6 mg/mL), no cytotoxic effects were observed after 24 h of exposure (Figure 1A). Cell viability remained above 90% at all concentrations, indicating that the formulation was well tolerated in hepatic cells even at the highest dose. As expected, treatment with the positive control (1 mg/mL SDS) resulted in complete loss of viability, validating assay performance.

#### 3.1.3. Glucose Uptake

Treatment with QUINOLAM significantly enhanced glucose uptake in HepG2 cells, in a dose-dependent manner (Figure 1B). Compared to untreated controls, cells exposed to 0.5 mg/mL, 1.0 mg/mL, and 2.5 mg/mL of QUINOLAM showed progressively higher uptake of the fluorescent glucose analog 2-NBDG. At the highest concentration (2.5 mg/mL), glucose uptake increased by more than 2-fold relative to baseline, suggesting a strong insulin-mimetic or insulin-sensitizing effect of the formulation.

#### 3.1.4. LDL-Receptor Expression

QUINOLAM upregulated LDL-R expression in HepG2 cells following 24 h exposure (Figure 1C). ELISA-based quantification revealed a dose-dependent increase in LDL-R protein levels at all tested concentrations (0.5, 1.0, and 2.5 mg/mL), with the highest dose eliciting the most pronounced response. These findings support a potential mechanism for LDL clearance enhancement through hepatic receptor-mediated uptake.

#### 3.1.5. Antioxidant Activity

In the TEAC assay, QUINOLAM exhibited potent antioxidant capacity (Figure 1D). Significant free radical scavenging activity was detected at concentrations of 0.1 mg/mL and above. The antioxidant effect was dose-dependent, with the formulation demonstrating strong Trolox-equivalent activity at 0.2 mg/mL. These results confirm the high antioxidant potential of the polyphenol-rich extract.

### 3.2. Clinical Findings

#### 3.2.1. Study Cohort

Of the 43 patients screened for eligibility, 30 met the predefined inclusion criteria and were included in the primary analysis. Thirteen individuals were excluded due to non-compliance with the standardized dosage regimen (2 tablets/day instead of 1 tablet/day). All included patients had completed a minimum of 12 weeks of continuous supplementation with the investigational product. Baseline characteristics of the study population are summarized in Table 1.

The study population consisted of adults with clinical and biochemical features consistent with metabolic syndrome. The mean age was 49.6 ± 9.6 years, and the mean BMI was 32.2 ± 6.1 kg/m^2^, indicative of class I obesity. The mean fasting plasma glucose was 103.8 ± 18,2 mg/dL, with 19 participants (63%) exhibiting values ≥100 mg/dL (subgroup mean: 114.2 ± 9.7 mg/dL). Mean total cholesterol and LDL-C were 219.1 ± 20.8 mg/dL and 146.5 ± 27.3 mg/dL, respectively, while HDL-C was within normal range (57.0 ± 14.1 mg/dL). Triglyceride levels averaged 113.7 ± 45.0 mg/dL. HbA1c was markedly elevated (8.89 ± 0.40%), indicating poor glycemic control in a substantial portion of the cohort. The mean HOMA-IR was 3.20 ± 1.19, consistent with significant insulin resistance, and CRP was 2.29 ± 1.5 mg/L, reflecting a low-grade inflammatory profile.

#### 3.2.2. Total Cholesterol

**Figure 2:**
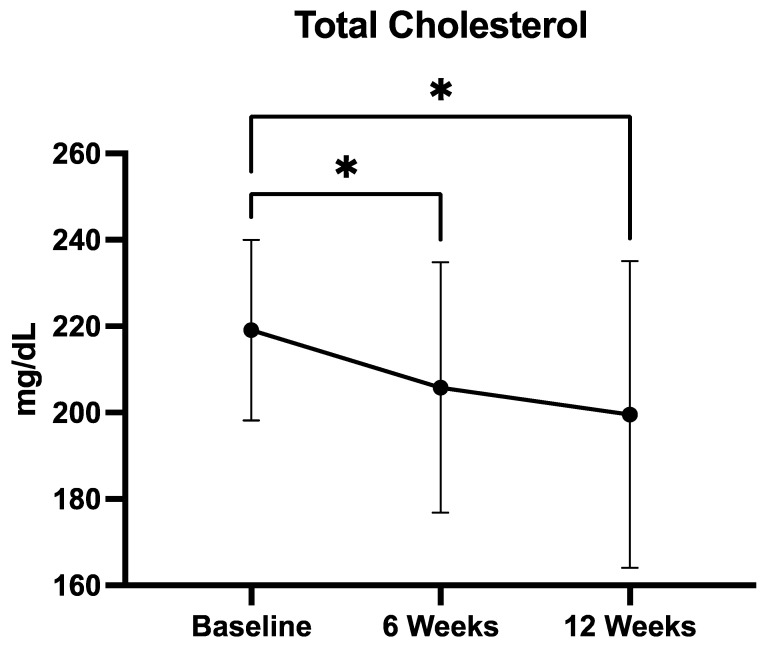
Mean Total Cholesterol levels at baseline, 6 weeks, and 12 weeks of supplementation with the QUINOLAM formulation in patients with metabolic syndrome (* *p* < 0.05).

Mean total cholesterol levels showed a progressive and statistically significant reduction over the 12-week supplementation period (Figure 2). Concentrations decreased from 219.1 ± 20.8 mg/dL at baseline to 205.6 ± 28.9 mg/dL at 6 weeks, and further to 199.5 ± 35.5 mg/dL at 12 weeks. The Friedman test confirmed a significant overall difference across timepoints (*p* = 0.0001). Post hoc analysis using Dunn’s multiple comparisons test revealed that both the reduction from baseline to week 6 (*p* < 0.05) and from baseline to week 12 (*p* < 0.05) were statistically significant. No significant difference was observed between week 6 and week 12. These findings indicate that the QUINOLAM formulation exerts a rapid and sustained cholesterol-lowering effect, with significant improvements evident as early as 6 weeks.

Total cholesterol decreased from 219.1 ± 20.8 mg/dL at baseline to 205.6 ± 28.9 mg/dL at week 6 and 199.5 ± 35.5 mg/dL at week 12. The overall change was statistically significant (Friedman test, *p* = 0.0001). Post hoc Dunn’s multiple comparisons test showed significant reductions from baseline to week 6 (*p* < 0.05) and from baseline to week 12 (*p* < 0.05), while the change from week 6 to week 12 was not significant. Data are presented as mean ± SD.

#### 3.2.3. LDL Cholesterol

**Figure 3:**
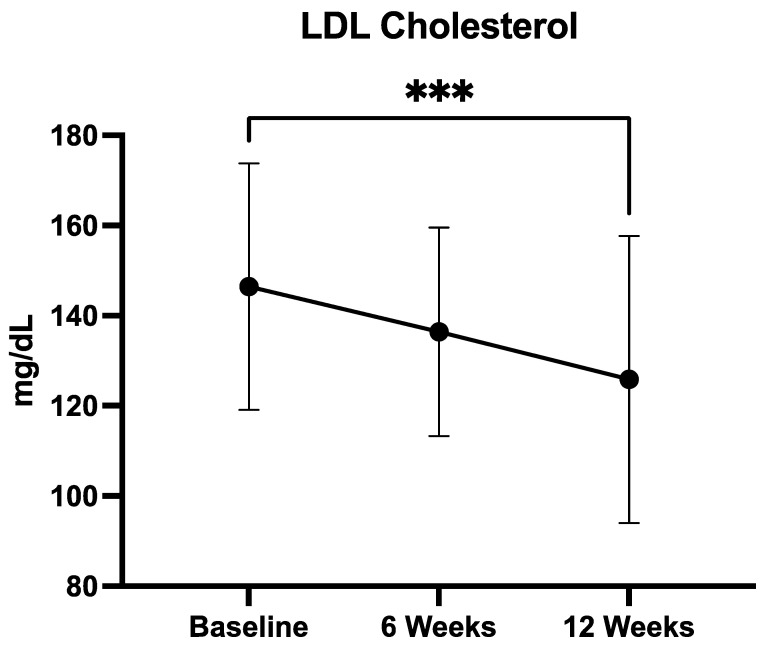
Mean LDL cholesterol (LDL-C) levels at baseline, 6 weeks, and 12 weeks of supplementation with the QUINOLAM formulation in patients with metabolic syndrome (*** *p* < 0.001).

Mean LDL cholesterol concentrations decreased over the course of supplementation, from 146.5 ± 27.3 mg/dL at baseline, to 136.4 ± 23.0 mg/dL at 6 weeks, and 125.9 ± 31.5 mg/dL at 12 weeks (Figure 3). Statistical analysis using the Friedman test showed a significant difference across the three timepoints (*p* = 0.0221), indicating an overall treatment effect. Post hoc analysis with Dunn’s multiple comparisons test confirmed a statistically significant reduction only between baseline and week 12 (*p* = 0.0156), while the differences between baseline and week 6, and between week 6 and week 12, did not reach significance. These findings suggest that sustained supplementation with the QUINOLAM formulation is required to achieve a clinically meaningful reduction in LDL cholesterol.

LDL-C decreased from 146.5 ± 23.0 mg/dL at baseline to 136.4 ± 23.0 mg/dL at week 6 and 125.9 ± 31.5 mg/dL at week 12. The overall change across timepoints was statistically significant (Friedman test, *p* = 0.0221), with post hoc Dunn’s test revealing a significant reduction only between baseline and week 12 (*** *p* < 0.001). Data are expressed as mean ± SD.

#### 3.2.4. Fasting Glucose

**Figure 4:**
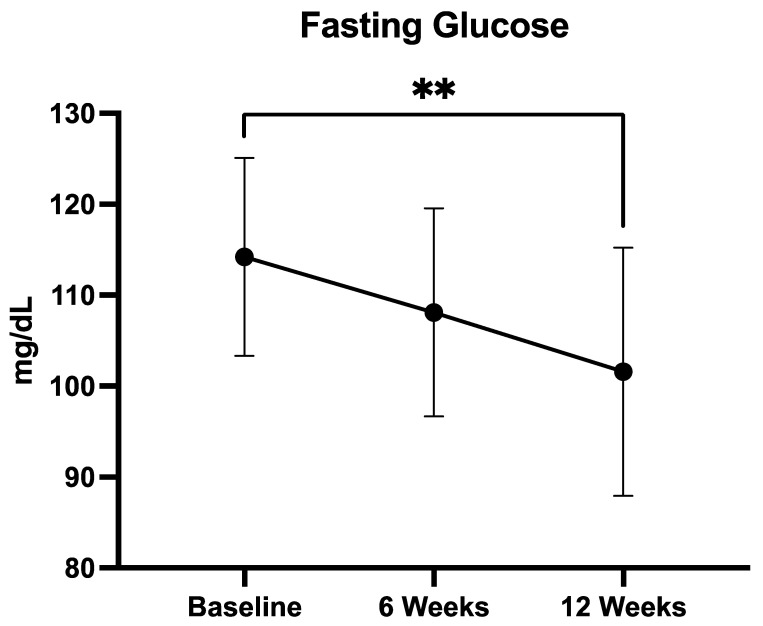
Mean fasting glucose levels at baseline, 6 weeks, and 12 weeks of supplementation with the QUINOLAM formulation in patients with metabolic syndrome and baseline glycemia ≥100 mg/dL (*n* = 19) (** *p* < 0.01).

In the predefined subgroup of patients with baseline fasting glucose ≥100 mg/dL (*n* = 19), mean glucose levels decreased from 114.2 ± 9.7 mg/dL at baseline to 108.2 ± 11.4 mg/dL at week 6, and 101.6 ± 13.6 mg/dL at week 12 (Figure 4). Statistical analysis with the Friedman test confirmed a significant overall difference across timepoints (*p* = 0.0021). Post hoc testing using Dunn’s multiple comparisons revealed a significant reduction from baseline to week 12 (*p* < 0.01), while changes from baseline to week 6 and from week 6 to week 12 were not statistically significant. These findings suggest that a clinically relevant glycemic improvement was achieved predominantly after 12 weeks of QUINOLAM supplementation.

Glucose levels decreased from 114.2 ± 9.7 mg/dL at baseline to 108.2 ± 11.4 mg/dL at week 6 and 101.6 ± 13.6 mg/dL at week 12. The overall reduction was statistically significant (Friedman test, *p* = 0.0021), with post hoc Dunn’s test showing a significant difference between baseline and week 12 (*p* < 0.01). Data are presented as mean ± SD.

#### 3.2.5. Insulin Resistance (HOMA-IR)

HOMA-IR values demonstrated a statistically significant decline between baseline and week 12 (Wilcoxon *p* = 0.024), indicative of improved insulin sensitivity. However, the Friedman test did not confirm significance across all timepoints (*p* = 0.104), suggesting variability in response or limited power for this endpoint.

### 3.3. Exploratory Findings

Several additional metabolic parameters demonstrated favorable trends, although these changes did not reach statistical significance. BMI showed a modest decrease at week 6; however, this reduction was not maintained at week 12, suggesting a transient effect on weight-related outcomes. HbA1c exhibited a slight downward shift over the study period, but the magnitude of change was insufficient to achieve statistical significance, possibly due to inter-patient variability or limited treatment duration.

Triglyceride concentrations decreased by approximately 14 mg/dL among participants with elevated baseline levels (≥100 mg/dL), yet the wide standard deviation associated with this change precluded statistical confirmation (*p* = 0.38). Similarly, CRP levels, while displaying a numerical reduction, did not significantly differ across timepoints (*p* = 0.15). These findings may reflect limited statistical power or heterogeneity in the inflammatory and metabolic response to the intervention, and should be regarded as descriptive trends that may inform the selection of endpoints and sample size calculations for future prospective studies, rather than as evidence of a definitive supplement effect.

## 4. Discussion

In this retrospective analysis of patients with MetS, a 12-week supplementation with a standardized nutraceutical blend (QUINOLAM), combining *Amaranthus* spp., *Cydonia oblonga* (quince), and *Olea europaea* (olive leaf) extracts, resulted in statistically significant reductions in total cholesterol, as well as improvements in LDL-C, fasting glucose (among dysglycemic individuals), and insulin resistance. These findings support the metabolic efficacy of each botanical component and extend their relevance to a real-world clinical setting. Nonetheless, while these results are consistent with prior literature on the individual components, the retrospective, non-controlled design of the study precludes any causal attribution of the observed changes solely to QUINOLAM.

Our primary outcome mirrors previous clinical findings. An RCT investigated the metabolic effects of amaranth oil in obese adults during a calorie-restricted diet [29]. Compared to rapeseed oil, amaranth oil significantly reduced total cholesterol and LDL-C, fasting glucose, and HOMA-IR, suggesting a potent lipid- and glucose-modulating effect in humans with metabolic disturbances [29]. Conversely, a crossover trial by Dus-Zuchowska et al. found that amaranth oil unexpectedly increased LDL-C in overweight individuals, likely due to the type of oil used, its fatty acid composition, and participant baseline metabolic status [30]. These heterogeneous findings underscore the importance of formulation, population characteristics, and contextual dietary factors in shaping the lipid outcomes of plant-based interventions. With regard to quince, clinical trials remain limited but promising. Notably, our findings further support the lipid-lowering potential of quince in a mixed population with documented metabolic syndrome, in line with this emerging evidence base. Olive leaf extract has been less frequently studied in combination with nutraceuticals but is well supported by both mechanistic and clinical evidence. Rich in phenolic secoiridoids such as oleuropein and hydroxytyrosol, the olive leaf extract has demonstrated potent antioxidant, anti-inflammatory, and vasoprotective activity [31,32]. Human trials have shown that olive leaf extract improves insulin sensitivity and glycemic control in overweight and prediabetic individuals, reduces blood pressure in hypertensive patients, and lowers LDL-C and total cholesterol in both normolipidemic and dyslipidemic cohorts [19,20,33,34,35,36].

Mechanistically, the efficacy of QUINOLAM is also supported by preclinical findings. Our in vitro analyses demonstrated that QUINOLAM enhanced glucose uptake in HepG2 hepatic cells in a dose-dependent manner, increased LDL receptor expression, and exerted potent antioxidant activity at physiologically relevant concentrations. These effects are consistent with known mechanisms of the individual extracts. Indeed, amaranth is rich in squalene, tocopherols, polyunsaturated fatty acids, and peptides with bioactive properties [37]. Studies with animals demonstrated that amaranth squalene reduces hepatic cholesterol synthesis and increases fecal cholesterol excretion in hyperlipidemic rats, and that amaranth oil downregulates HMG-CoA reductase, the rate-limiting enzyme in endogenous cholesterol biosynthesis [38,39]. In vitro studies confirmed that peptides derived from *Amaranthus* spp. inhibit HMG-CoA reductase and exhibit ACE-inhibitory activity, with implications for both lipid and blood pressure control [40,41]. These effects may collectively explain the consistent reductions in total and LDL-C observed in our study.

Parallel lines of evidence support the metabolic actions of quince. Polyphenol-rich extracts of quince have demonstrated robust antioxidant activity and anti-adipogenic effects in murine 3T3-L1 cell models, with in vivo studies showing reductions in total cholesterol, triglycerides, LDL cholesterol, and adipose tissue mass in diet-induced obese mice [42]. Measured bioactive compounds such as 5-O-caffeoylquinic acid, proanthocyanidins, and flavonoids exert anti-inflammatory effects through modulation of NF-κB signaling and promotion of insulin-mediated glucose uptake via the PI3K/AKT pathway [43]. Additionally, quince polyphenols influence the gut microbiome, leading to secondary metabolites that enhance systemic metabolic outcomes [44].

In aged rodent models, olive leaf extract supplementation reduces serum LDL-C and IL-6, enhances hepatic antioxidant enzymes (e.g., SOD-1, GSR), and preserves endothelial nitric oxide-dependent vasodilation [18,45]. These findings are highly relevant in MetS, where endothelial dysfunction and oxidative stress are central to disease progression. The observed upregulation of LDL receptor expression in HepG2 cells exposed to QUINOLAM suggests that the olive leaf extract may enhance hepatic LDL clearance through post-transcriptional mechanisms, as previously proposed in human and animal studies. Despite the clear benefits provided by each individual component of QUINOLAM, we acknowledge that the concentrations used in our in vitro experiments (0.5–2.5 mg/mL) exceed those achievable through oral supplementation in vivo, limiting the direct translational applicability of these mechanistic findings.

Another potential pathway through which QUINOLAM supplementation could exert metabolic effects is modulation of the gut microbiome, as several polyphenol-rich plant extracts have been shown to influence microbial composition and metabolic activity [46,47]. Although this mechanism is plausible, it could not be evaluated in the present work because no microbiota assessments were performed as part of the original clinical management. Microbiome analysis requires dedicated prospective sampling and specialized laboratory techniques, which are outside the scope of retrospective observational studies. Future controlled investigations specifically designed for mechanistic exploration should include microbiome profiling to clarify whether any of the observed metabolic changes are mediated by gut microbial modulation. Another unmeasured but potentially relevant factor is the contribution of lifestyle variables such as adherence to a Mediterranean diet, physical activity, sleep quality, and alcohol or tobacco use. These were not recorded in the clinical charts and therefore could not be analyzed, representing a limitation inherent to retrospective medical record reviews.

Apart from these considerations, our data suggest that the combination of *Amaranthus*, *Cydonia oblonga*, and *Olea europaea* may exert complementary and potentially synergistic effects on key metabolic pathways. Specifically, these extracts appear to influence lipid metabolism, glycemic control, and inflammatory responses, which are three major pathophysiologic indicators of metabolic syndrome. By targeting multiple mechanisms simultaneously, this formulation may provide broader benefits than any single extract alone, supporting its potential role as a nutritional strategy in metabolic risk management.

Nonetheless, several limitations must be considered when interpreting our findings. Most importantly, the observational and non-randomized nature of the study prevents causal inference, and the findings should be interpreted as associations. Without a randomized comparator group, the observed changes could be partially attributed to unmeasured confounding variables such as concurrent lifestyle modifications, changes in diet, or physical activity. Although the sample size was sufficient to detect changes in the primary endpoint, it may have been underpowered to identify smaller yet clinically relevant shifts in secondary outcomes such as HbA1c, triglycerides, or inflammatory markers. Moreover, the intervention period of 12 weeks, while adequate for lipid assessment, may have been too short to capture long-term metabolic adaptations, particularly regarding weight or glycemic durability. Also, the exclusion of patients who did not adhere to the standard supplement regimen improved internal validity but may limit generalizability to broader populations. Furthermore, we did not collect data on mechanistic biomarkers, which would have enriched our understanding of physiological responses to treatment. Finally, the single-center nature of the study and the exclusion of non-adherent patients limit external validity; however, these exclusions were necessary to preserve internal consistency and ensure reliable evaluation of the intervention. Stratified analyses by age, sex, baseline therapy, or comorbidities were not performed, as the small sample size would have rendered such analyses underpowered and uninterpretable. Instead, we focused on the overall population-level trends.

Despite these limitations, the study offers clinically relevant insights. It provides real-world evidence that a botanically derived supplement containing QUINOLAM may beneficially modulate key metabolic syndrome components in patients not receiving lipid-lowering pharmacotherapy. The concordance between our findings and previous animal and human studies enhances the biological plausibility and supports the rationale for future prospective, controlled trials that integrate lifestyle assessments, microbiome profiling, and comprehensive safety monitoring.

## 5. Conclusions

This retrospective observational study provides preliminary evidence that supplementation with a standardized combination of *Cydonia oblonga* (quince), *Olea europaea* leaves, and *Amaranthus* spp. may exert clinically meaningful effects on lipid and glucose metabolism in patients with metabolic syndrome. Specifically, the formulation was associated with a significant reduction in total cholesterol and LDL-C, as well as favorable changes in fasting glucose and insulin resistance among dysglycemic individuals. These outcomes, although associative and not causal, align with existing mechanistic and clinical data suggesting that the bioactive constituents of quince, olive leaves and amaranth may modulate key metabolic pathways involved in dyslipidemia, insulin sensitivity, and systemic inflammation. Importantly, these benefits were observed in a real-world clinical setting, in the absence of lipid-lowering pharmacotherapy, supporting the relevance of such nutraceuticals as adjunctive options for patients with elevated metabolic risk.

Nonetheless, caution is warranted in interpreting these findings due to the non-randomized design, limited sample size, and relatively short follow-up duration. Larger, prospective, placebo-controlled trials are needed to confirm efficacy, evaluate safety, and explore the mechanistic underpinnings of the observed effects. If further validated, this botanical formulation could represent a valuable, low-burden intervention in the broader strategy for cardiometabolic risk reduction.

## Figures and Tables

**Table 1:** Baseline characteristics of the study population. This table summarizes the demographic, anthropometric, and biochemical parameters of the patients included in the primary analysis. Values are presented as mean or frequency ± SD, as appropriate. FPG: fasting plasma glucose; LDL-C: low-density lipoprotein cholesterol; HDL-C: high-density lipoprotein cholesterol; HbA1c: glycated hemoglobin; HOMA-IR: Homeostasis Model Assessment of Insulin Resistance; CRP: C-reactive protein.

	Value
Patients screened	43
Patients included in analysis	30
Excluded due to non-compliance	13
Age (±years)	49.6 ± 9.6
BMI (±kg/m^2^)	32.2 ± 6.1
Mean Fasting Plasma Glucose (FPG) (±mg/dL)	103.8 ± 18.2
FPG ≥ 100 mg/dL (*n*, %)	19 (63%)
FPG ≥ 100 mg/dL (mean, ±mg/dL)	114.2 ± 9.7
Total cholesterol (mg/dL)	219.1 ± 20.8
LDL-C (±mg/dL)	146.5 ± 27.3
HDL-C (±mg/dL)	57.0 ± 14.1
Triglycerides (±mg/dL)	113.7 ± 45.0
HbA1c (±%)	8.89 ± 0.40
HOMA-IR	3.20 ± 1.19
CRP (±mg/L)	2.29 ± 1.5

## Data Availability

The data presented in this study are available on request from the corresponding author due to privacy reasons.

## Abbreviations

The following abbreviations are used in this manuscript:

MetS	Metabolic syndrome
RCTs	Randomized controlled trials
QUINOLAM	Quince, olive leaf, and amaranth
GLP	Good Laboratory Practices
HPLC-PDA	High-performance liquid chromatography with photodiode array detection
ADF	Acid detergent fiber
NDF	Neutral detergent fiber
FBS	Fetal bovine serum
SDS	Sodium dodecyl sulfate
HDL-C	High-density lipoproteins cholesterol
LDL-C	Low-density lipoproteins cholesterol
HOMA-IR	Homeostasis Model Assessment of Insulin Resistance
TEAC	Trolox Equivalent Antioxidant Capacity
CRP	C-reactive protein
BMI	Body mass index
CRF	Case report form
GDPR	General Data Protection Regulation
